# Demographic and clinical characteristics of foreign nationals accessing psychiatric services in Japan: a multicentre study in a metropolitan area

**DOI:** 10.1186/s12888-020-02951-z

**Published:** 2020-12-03

**Authors:** Youji Takubo, Takahiro Nemoto, Momoko Iwai, Minako Kashima, Eriko Yamaguchi, Akiko Maruyama, Sachio Miura, Hisaaki Saito, Naohisa Tsujino, Masafumi Mizuno

**Affiliations:** 1 Department of Neuropsychiatry, Toho University Graduate School of Medicine, 5-21-16 Omori-nishi, Ota-ku, Tokyo, 143-8540 Japan; 2 Department of Psychiatry, Saiseikai Yokohamashi Tobu Hospital, 3-6-1 Shimosueyoshi, Tsurumi-ku, Yokohama, Kanagawa 230-8765 Japan; 3 Department of Neuropsychiatry, Toho University Faculty of Medicine, 6-11-1 Omori-nishi, Ota-ku, Tokyo, 143-8541 Japan; 4 Department of Neuropsychiatry, Kawasaki Municipal Hospital, 12-1 Shinkawadori, Kawasaki-ku, Kawasaki, Kanagawa 210-0013 Japan; 5 Shoin University Faculty of Nursing, 9-1 Morinosato-wakamiya, Atsugi, Kanagawa 243-0124 Japan; 6 Nagasaki University, 1-12-4 Sakamoto, Nagasaki, Nagasaki 852-8523 Japan; 7 NPO MAIKEN, 2-1935-7 Motohachioji-machi, Hachioji, Tokyo, 193-0826 Japan

**Keywords:** Acculturation, Foreign nationals, Immigrants, Immigration, Japan, Medical interpreter, Mental health

## Abstract

**Background:**

International immigration to Japan, where homogeneous ethnicity is a population characteristic, has been growing. Although immigration is recognised as a risk factor for multiple mental-health related issues, there are few regional reports on foreign nationals accessing the psychiatric services in Japan. We aimed to reveal their current status and provide information to develop an optimal service system.

**Methods:**

A multicentre retrospective document review research was conducted. The subjects were foreign nationals who resided in Japan and presented at the psychiatry departments in three core regional hospitals in the Keihin region, which faces Tokyo Bay and is well known to include the largest traditional industrial zone in Japan, over a period of 3 years. We investigated the patients’ demographic and clinical information including country/region of origin, spoken language, use of a medical interpreter, pathway to hospitals and outcome.

**Results:**

The percentage of foreign patients among all patients (1.4%) was quite low. Their age distribution (45.8 years on average) was dissociated from the age distribution of foreign nationals who resided in Japan. Regarding the country/region of origin, China (35.1%) was the most common country, followed by the Philippines, Korea and Brazil. Several subjects (22.9%) could not speak Japanese; therefore, interpretation was required by family members/friends (17.1%) or a professional interpreter (5.4%). Neurotic and stress-related disorders were the most common diagnosis (24.4%). The proportion of psychoactive substance use was higher than that for Japanese national data as immigrants are known to be at risk for it.

**Conclusions:**

The results suggest that foreign nationals who reside in Japan are less likely to contact appropriate services for mental illness, especially young people at relatively high risk of mental illness do not access services. Furthermore, lack of medical interpreters may impede the mental health conditions of foreign nationals. The development of a community-based integrated care system accessible to foreign nationals seems to be indispensable.

## Background

International immigration has been increasingly recognised as an important issue in modern society, and the worldwide number of immigrants is growing [1]. Castles et al. [2] mentioned that globalisation and differentiation were characteristics of recent international immigration. Host countries need to accept diverse foreign nationals with economically, socially, and culturally different backgrounds [3].

Immigration to Japan, where homogeneous ethnicity is a population characteristic, has been growing in the last three decades after the reorganisation of the resident status of foreign nationals in 1990 [4, 5]. The Japanese government has accepted foreign nationals as manpower mainly from Asian countries because of the extreme aging of society and resulting labour shortage. According to the 2017 statistics regarding the inflow of foreign populations into countries belonging to the Organisation for Economic Cooperation and Development (OECD), the inflow to Japan is 475,000 people per year and is the fourth highest among these countries [6]. The Ministry of Justice of Japan reported that the number of foreign nationals who resided in Japan was over 2.82 million in 2019, accounting for 2.24% of the total population in Japan [7, 8]. Among foreign nationals who reside in Japan, the largest groups are from China (0.79 million people), followed by Korea (0.45 million), Vietnam (0.37 million), the Philippines (0.28 million) and Brazil (0.21 million) [7]. An amendment to the Immigration Control and Refugee Recognition Act in 2019 created a new residence status called “Specified Skilled Worker”, which is a residence status for foreign nationals engaged in work requiring skills that require considerable knowledge or experience in a specified industrial field [9]. Therefore, more foreign nationals are expected to begin working stably in Japan [5].

Immigration is a stressful experience that involves significant obstacles in many aspects of individual lives [10–12]. Immigrants face cultural distances in their new society, such as difficulties accessing various social resources as well as language problems [13]. Accordingly, immigration has also been recognised as a socio-economic burden that influences general and mental health [14–16]. Immigrants are likely to experience psychological distress, called “acculturative stress”, during the process of cultural adaptation [17]. Although there are different coping styles and resiliencies to acculturative stress among immigrant groups [12], immigrants usually feel strong distress during the first 5 years after immigration [14, 18, 19]. Previous studies have reported the vulnerability of immigrants and refugees to mental ill-health [10, 14–16]. Immigrants’ distress in their daily lives is thought to cause various psychiatric symptoms [10], and immigration is recognised as a risk factor for psychiatric disorders such as stress-related disorders, mood disorders, substance abuse and psychoses [11, 15, 20, 21]. Systematic reviews and meta-analyses have revealed that the incidence of psychotic disorders among immigrants and ethnic minority populations is about 1.5 to 3.0 times as high as that in ethnic majority populations [15]. Whereas a meta-analysis did not show a significant increase in mood disorders associated with immigration [22], immigrants who had been diagnosed as having depression were more likely to experience suicidal ideation [23]. Moreover, recent studies have shown that children and adolescents in immigrant families experience severe acculturative stress, which is associated with poor trajectories in mental health such as alcohol and substance use, eating disorders, and emotional and psychological problems [18, 24].

Prejudice and discrimination related to immigrants are also a critical problem [25, 26]. A survey conducted by the Ministry of Justice of Japan reported that 30% of foreign nationals experienced discrimination, 40% were declined residence, and 25% were not employed because of their nationality [27]. Although immigration may have a negative impact on mental health [12, 14, 16, 28], previous studies examining the use of primary services for mental health problems by immigrants found that immigrants were less likely to contact such services, compared with the majority populations [13, 29]. Furthermore, a survey showed that immigrants had had significantly less contact with primary health care services at both 1 month and 6 months before their suicide [30].

In Japan, large numbers of foreign nationals are concentrated in metropolitan areas such as Tokyo, Aichi, Osaka and Kanagawa. The number of foreign nationals in these four prefectures has increased to 47% of foreign nationals across Japan [7]. The Keihin region covers the southeastern Tokyo area, southern Kawasaki, and eastern Yokohama area in Kanagawa Prefecture [31]. The Keihin region faces Tokyo Bay and is well known to include the largest traditional industrial zone in Japan. There are 112,000 foreign nationals in the Keihin region [7]; however, little information is available on their mental health. Although there has been a systematic review of the mental well-being of international immigrants to Japan [28], the subjects in the review were not a clinical sample but were instead members of the general population, such as students and workers. To the best of our knowledge, few regional studies using clinical samples of foreign nationals with mental health problems have been conducted in Japan, even if reports written in Japanese were taken into consideration [32–35]. At present, only a few medical institutions support mental health care for foreign nationals in Japan. According to a national survey, whereas 80% of Japanese medical institutions accepted foreign patients, only 13% of institutions had experience using medical interpreters [36]. Given the increasing number of foreign nationals in Japan, the need to provide integrated care has been emphasised. However, there is a lack of data regarding this issue in Japan. In order to reveal the current status of foreign nationals’ mental health and provide information to develop an optimal community-based integrated care system that includes early intervention and considers foreign nationals, we investigated the demographic and clinical characteristics of foreign nationals accessing the psychiatric services in a metropolitan area (Keihin region).

## Methods

### Procedures and subjects

This is a retrospective document review research. The subjects were foreign nationals who resided in Japan and presented at the psychiatry departments of three central hospitals in the Keihin region over a three-year period from April 1, 2016, to March 31, 2019. Of the three hospitals, the Toho University Omori Medical Centre (TUO) covers the southeastern area of Tokyo, the Kawasaki Municipal Hospital (KMH) covers the southern area of Kawasaki, and the Saiseikai Yokohamashi Tobu Hospital (SYT) covers the eastern area of Yokohama. We investigated the patients’ demographic and clinical information including country/region of origin, spoken language, family members, use of a medical interpreter, pathway to hospitals, medical history, substance use, and follow-up and outcome. Psychiatric diagnoses were based on the criteria of the International Statistical Classification of Diseases and Related Health Problems, 10th Revision (ICD-10) [37].

This study was performed as part of the Mental health and Early Intervention in the Community-based Integrated care System (MEICIS) project supported by the Health Labour Sciences Research Grant (19GC1015).

The study protocol was approved by the Ethics Committees of the Faculty of Medicine, Toho University (A19058), KMH (2019–21), and SYT (2019010). Informed consent was obtained in the form of opt-out on a website. The study was performed in accordance with the latest version of the Declaration of Helsinki (October 2013).

### Analysis

For comparisons of the data obtained in the present study with the nationwide circumstances in Japan, national survey data on foreign nationals who resided in Japan [7], population in Japan [8] and patients with psychiatric disorders [38] were used.

## Results

A total of 205 individuals’ documents (1.4%) met the inclusion criteria among all 14,511 patients who visited the psychiatry departments of three hospitals, consisting of 62 foreign nationals out of 7269 patients (0.8%) at TUO, 91 out of 3566 (2.6%) at KMH, and 52 out of 3649 (1.4%) at SYT (Table 1).

**Table 1:** Demographics and clinical information for foreign patients

	Total *n* = 205		TUO *n* = 62		KMH *n* = 91		SYT *n* = 52	
	n	%	n	%	n	%	n	%
Sex								
Male	72	35.1	29	46.8	28	30.8	15	28.8
Female	133	64.9	33	53.2	63	69.2	37	71.2
Age (mean, SD)	45.8	16.5	46.1	18.3	46.7	16.0	43.9	14.7
**Country/region of origin**								
China	72	35.1	27	43.5	33	36.3	12	23.1
Philippines	38	18.5	11	17.7	19	20.9	8	15.4
Korea	33	16.1	5	8.1	16	17.6	12	23.1
Brazil	10	4.9	0	0.0	6	6.6	4	7.7
United States	7	3.4	1	1.6	2	2.2	4	7.7
Taiwan	7	3.4	4	6.5	2	2.2	1	1.9
Vietnam	5	2.4	3	4.8	1	1.1	1	1.9
Peru	4	2.0	0	0.0	2	2.2	2	3.8
India	3	1.5	1	1.6	0	0.0	2	3.8
Bangladesh	3	1.5	0	0.0	2	2.2	1	1.9
Others	23	11.2	10	16.1	8	8.8	5	9.6
**Language**								
Cannot speak Japanese	47	22.9	17	27.4	14	15.4	16	30.8
Interpreted by relatives	35	17.1	16	25.8	11	12.1	8	15.4
Interpreted by a professional	11	5.4	1	1.6	2	2.2	8	15.4
**Pathway to hospital visitation**								
Voluntary visit	89	43.4	10	16.1	56	61.5	23	44.2
From other hospitals	52	25.4	19	30.6	25	27.5	8	15.4
From other departments	42	20.5	23	37.1	4	4.4	15	28.8
Suicide attempt	8	3.9	4	6.5	1	1.1	3	5.8
Request of the police	6	2.9	0	0.0	5	5.5	1	1.9
From the RHQ	2	1.0	2	3.2	0	0.0	0	0.0
Temporary visit on parole	2	1.0	0	0.0	0	0.0	2	3.8
Others	4	2.0	4	6.5	0	0.0	0	0.0
**Diagnosis**								
F0	12	5.9	7	11.3	2	2.2	3	5.8
F1	13	6.3	4	6.5	6	6.6	3	5.8
F2	42	20.5	12	19.4	20	22.0	10	19.2
F3	41	20.0	12	19.4	14	15.4	15	28.8
F4	50	24.4	14	22.6	23	25.3	13	25.0
F5	22	10.7	1	1.6	15	16.5	6	11.5
F6	3	1.5	0	0.0	2	2.2	1	1.9
F7	4	2.0	1	1.6	3	3.3	0	0.0
F8	1	0.5	1	1.6	0	0.0	0	0.0
F9	3	1.5	3	4.8	0	0.0	0	0.0
G40	8	3.9	1	1.6	6	6.6	1	1.9
No diagnosis	6	2.9	6	9.7	0	0.0	0	0.0
**Substance use**								
Alcohol	11	5.4	4	6.5	5	5.5	2	3.8
Amphetamine	5	2.4	0	0.0	2	2.2	3	5.8
Cannabis	3	1.5	0	0.0	0	0.0	3	5.8
Thinner	2	1.0	0	0.0	1	1.1	1	1.9
Others	3	1.5	3	4.8	0	0.0	0	0.0
**Treatment**								
Outpatient	194	94.6	60	96.8	83	91.2	51	98.1
Outpatient after hospitalisation	7	3.4	2	3.2	5	5.5	0	0.0
Hospitalisation	4	2.0	0	0.0	3	3.3	1	1.9
**Outcome**								
Followed up	90	43.9	26	41.9	39	42.9	25	48.1
Discontinued by oneself	66	32.2	21	33.9	26	28.6	19	36.5
Finished follow-up	22	10.7	5	8.1	12	13.2	5	9.6
Introduced to other hospitals/clinics	21	10.2	8	12.9	11	12.1	2	3.8
Only hospitalisation	3	1.5	0	0.0	2	2.2	1	1.9

*TUO* Toho University Omori Medical Centre, *KMH* Kawasaki Municipal Hospital, *SYT* Saiseikai Yokohamashi Tobu Hospital, *RHQ* Refugee Assistance Headquarters, *F0* Organic, including symptomatic, mental disorders, *F1* Mental and behavioral disorders caused by psychoactive substance use, *F2* Schizophrenia, schizotypal and delusional disorders, *F3* Mood (affective) disorders, *F4* Neurotic, stress-related and somatoform disorders, *F5* Behavioural syndromes associated with physiological disturbances and physical factors, *F6* Disorders of adult personality and behaviour, *F7* Mental retardation, *F8* Disorders of psychological development, *F9* Behavioural and emotional disorders with onset usually occurring in childhood and adolescence (F90-F98), *G40* Epilepsy

**Fig. 1:**
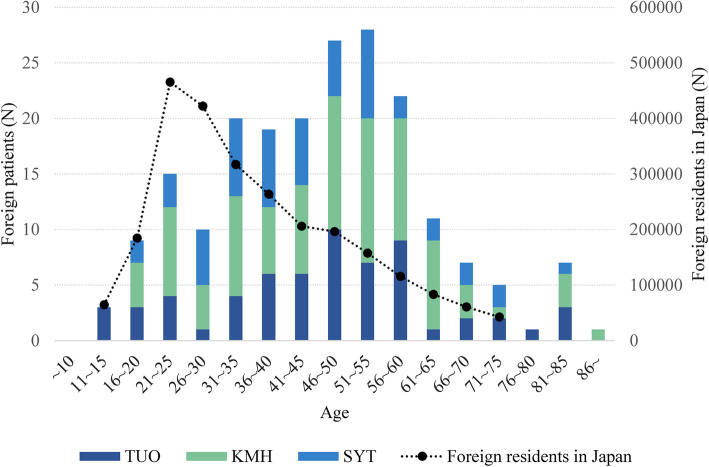
Age distributions of foreign patients visiting three hospitals and foreign nationals living in Japan. The number of foreign nationals between the ages of 11–75 years living in Japan (foreign national statistics as of June 2019 [7]) is shown by the dotted line on the bar graph, which shows the ages of the foreign patients visiting three hospitals. TUO: Toho University Omori Medical Centre; KMH: Kawasaki Municipal Hospital; and SYT: Saiseikai Yokohamashi Tobu Hospital

The overall sex ratio among the foreign patients was 1:1.9 (men:women), and the mean age was 45.8 years. The distribution of foreign patients according to age is shown in Fig. 1, with the number of foreign nationals who reside in Japan shown by the dotted line [7].

Regarding the country/region of origin, China (35.1%) was the most common, followed by the Philippines (18.5%), Korea (16.1%) and Brazil (4.9%). Forty-seven (22.9%) of the 205 subjects could not speak Japanese; therefore, 35 subjects (17.1%) required interpretation by their family members or friends, and 11 subjects (5.4%) required interpretation by a professional interpreter.

Regarding the pathways to hospital visitation, voluntary visits by themselves or at the recommendation of family or friends (43.4%) were the most common, followed by introductions from other hospitals (25.4%) and introductions from other departments within the same hospital (20.5%). Eight patients (3.9%) visited because of suicide attempts, and six patients (2.9%) visited at the request of the police.

**Fig. 2:**
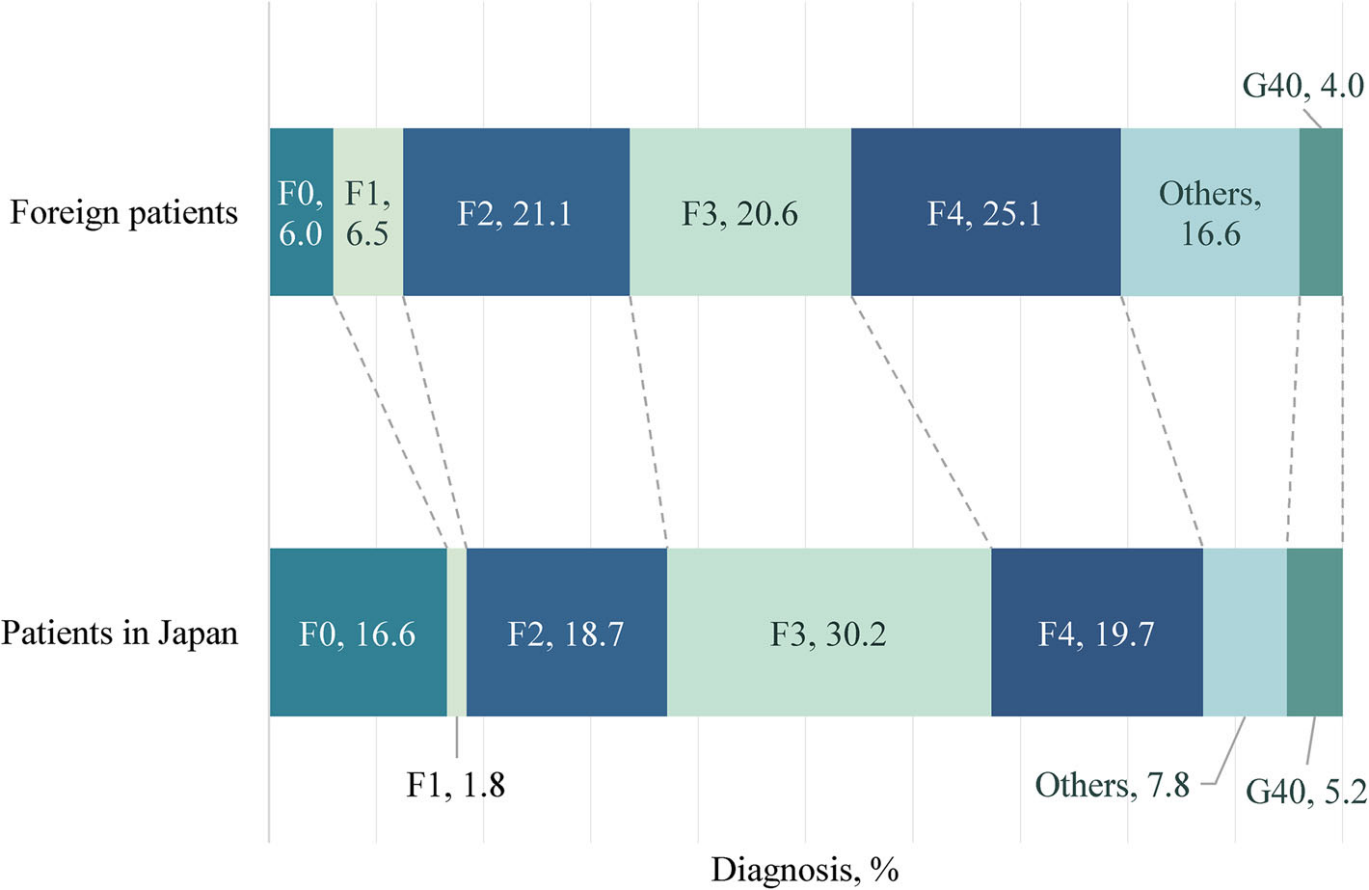
Comparison of diagnoses among foreign patients visiting three hospitals and data from a national survey. Psychiatric patients in Japan: number of patients with mental illness from a “Patient Survey” conducted in 2017 [38]. F0: Organic, including symptomatic, mental disorders; F1: Mental and behavioural disorders caused by psychoactive substance use; F2: Schizophrenia, schizotypal and delusional disorders; F3: Mood (affective) disorders; F4: Neurotic, stress-related and somatoform disorders; G40: Epilepsy. Other diagnoses included behavioural syndromes associated with physiological disturbances and physical factors (ICD-10 code: F5), disorders of adult personality and behaviour (ICD-10 code: F6), mental retardation (ICD-10 code: F7), disorders of psychological development (ICD-10 code: F8), and behavioural and emotional disorders with onset usually occurring in childhood and adolescence (ICD-10 code: F90-F98)

From the viewpoint of diagnosis, neurotic, stress-related and somatoform disorders (ICD-10 code: F4) were the most common (24.4%), followed by schizophrenia, schizotypal and delusional disorders (ICD-10 code: F2) (20.5%) and mood disorder (ICD-10 code: F3) (20.0%). The proportion of diagnoses was compared with Japanese national data to investigate the mental health problems that are likely to occur in foreign nationals who reside in Japan [38]. In the comparison, six subjects who had consulted a psychiatrist at TUO for a routine examination before undergoing renal transplantation but who had not received a diagnosis were excluded (Fig. 2).

Regarding treatment, only a few patients were hospitalized (5.4%); 90 patients (43.9%) received continuous follow-up care at an outpatient department, and 66 patients (32.2%) discontinued their treatment by themselves.

## Discussion

We tried to clarify the characteristics of foreign nationals visiting psychiatric departments in the Keihin region, which is the largest metropolitan area of Japan. The results revealed that the proportion of foreign patients was 1.4% among the total patients, that most of the foreign nationals were from Asian countries, and that their mean age was 45.8 years. We also revealed that there were about twice as many female patients as there were male patients, and that neurotic disorders (ICD-10 code: F4) were the most common diagnosis. Foreign patients who could not speak Japanese received interpretation through a family member or friend more frequently than through a trained medical interpreter.

### Country/region of origin

The percentage of foreign nationals accessing the psychiatric services (1.4%) was relatively low, compared with the percentage of foreign nationals living in the Keihin region (4.4%) [7, 8]. This result suggests that foreign nationals in Japan are less likely to contact adequate services for mental health problems, compared with the majority of the population, similar to the results of previous studies reported for other countries [13, 29]. Whereas Japan has the National Health Insurance System that all residents in Japan, which include foreign nationals, are obligated to join, various socioeconomic status may affect their access to services [29, 39]. In addition to these circumstances, further research is needed to address confounding factors such as internalised stigma and the resilience of immigrants.

The distribution of nationalities in the present study was similar to that of foreign nationals living in Japan overall (Table 1). When looking at changes in foreign populations over the past 10 years in Japan, the numbers of Chinese and Filipinos have been increasing moderately, while the number of Koreans has been gradually decreasing. Notably, the number of Vietnamese is growing rapidly, increasing from 41,000 in 2009 to 370,000 in 2019 [7]. Of the 110,000 foreign nationals living in the Keihin region, 12,000 are Vietnamese, and the Keihin region is known to be an area where the number of Vietnamese is increasing rapidly [7]. Given that this increase in Vietnamese people living in the Keihin region is relatively new, the low proportion of Vietnamese patients in the present study may suggest that a short duration of residing in Japan as an ethnic group may be a barrier to appropriate consultation [28, 40]. Furthermore, many Vietnamese individuals living in Japan are young technical intern trainees, and this characteristic seems to be related to a relatively short visit [4].

### Distribution of foreign patients according to age

The age distribution of the foreign national population in Japan peaks at individuals in their twenties, which corresponds to an age of increased susceptibility to mental illnesses (Fig. 1) [7, 41, 42]. Since immigrants are known to have greater mental vulnerability than the majority populations in a community [10, 15, 16, 23], a number of foreign nationals in Japan are thought to be at a high risk of mental illness. Regarding residence status, the number of foreign students and technical intern trainees is rapidly increasing, accounting for 24% of the total for foreign nationals in 2019 [7]. This means that the inflow of foreign nationals in their youth or early adulthood has increased in Japan. Mental health problems in young adults negatively impact academic, professional and social activities [43]. While the distribution of psychiatric patients by age generally follows the distribution of the total population by age in Japan [38, 44], the age distribution of foreign nationals who visited psychiatric department at three hospitals was dissociated from the age distribution of foreign nationals who resided in Japan (Fig. 1) [7]. This suggests that young foreign nationals with a relatively high risk for mental illness are not accessing appropriate services. The lack of access among young foreign nationals may also be explained by a short-period of residence in Japan [13, 28], although further investigations are needed. On the other hand, the fact that middle-aged patients were prominent in the present study may be explained by the association of these individuals with long-term residents who may face fewer language barriers [13, 28]. A previous report showed that a deficiency in social connections in post-migratory surroundings can lead to isolation and distress [40]. Therefore, there is a need to develop community-based integrated mental health services that consider foreign nationals [13, 42, 45].

### Sex ratio of foreign patients

The sex ratio of psychiatric patients in Japan was almost even in a national survey [38]. However, the number of male patients in the present study was disproportionally low, although the sex ratio of foreign nationals living in Japan is also almost even (males: 49.0%; females: 51.0%) [7]. This difference can be explained by a previous finding that male immigrants are known to be less likely to use services than female immigrants [13, 40]. Meanwhile, a systematic review on immigrants to Japan suggested that female immigrants faced barriers to mental well-being; however, most of the reviewed studies investigated specific samples, such as students [28].

### Pathways to hospital visitation and language problems

About 40% of the subjects visited the psychiatric departments voluntarily by themselves or at the recommendation of a family member or friend, and almost the same proportion of subjects were introduced by other hospitals or other departments in the same hospital. The result that the proportion of subjects introduced by other hospitals or departments was comparable with the proportion of subjects visiting on a voluntary basis is consistent with previous studies, indicating that immigrants may have difficulty seeking psychiatric medical consultations directly because of language barriers, a lack of encouragement from others or stigmas towards mental illness [28, 32, 46].

As for involuntary visits, the results that 3.9% of the patients visited because of suicide attempts and 2.9% visited at the request of the police also seem to be worth noting. Immigrants are reportedly more likely to experience suicidal ideation and to have received fewer services before a suicide [23, 30]. The present results also suggest that their mental health problems may not have been properly treated, resulting in suicide attempts. Regarding the subjects who visited at the request of the police, most of their diagnoses were schizophrenia. Further studies that examine the duration of untreated psychosis (DUP) among foreign nationals in Japan, who have difficulty accessing social support and resources, as well as among the total population in Japan are anticipated [47].

Regarding medical interpretation, a number of studies have reported that the quality of care in patients who did not speak a host country’s language was compromised when interpreters were not available, whereas trained professional interpreters have positive effects on patient satisfaction, quality of care and patient outcomes [48]. A systematic review on immigrants in Japan suggested two common barriers: troubles in communicating in Japanese, and a lack of social support [28]. Twenty-three percent of the subjects could not speak Japanese, and these subjects required interpretation by a family member, not a trained medical interpreter (Table 1). Professional medical interpreters are preferred because family interpreters can unknowingly convey technical errors because of a lack of expert knowledge. Inadequate interpretation may lead to serious consequences for patients with mental problems [48]. Language barriers are known to be associated with poor mental health, a low use of appropriate services and an increase in suicide behaviour [13, 28, 49]. The present study revealed that the use of medical interpreters remains rare, and this limitation may impede the health conditions, especially the mental health conditions, of foreign nationals.

### Diagnosis

Neurotic, stress-related and somatoform disorders (ICD-10 code: F4), which have a significant impact on social functioning [50], were the most common diagnoses (Table 1). The proportion of F4 diagnoses in this study was higher than that for Japanese national data (Fig. 2). This result suggests that acculturation stress in daily living surrounded by different cultures and habits affects foreign nationals, as previous studies have reported [10, 17]. The proportion of schizophrenia, schizotypal and delusional disorders (ICD-10 code: F2), which was the second most common diagnosis in this study, was almost equal to that for Japanese national data (Fig. 2). Immigration is reportedly a risk factor for the onset of psychosis [15]. The proportion of F2 diagnoses in this study would likely be higher if young foreign nationals visited hospitals when needed. Mood disorder (ICD-10 code: F3) was the third most common diagnosis, and the proportion of F3 diagnoses in this study was relatively smaller than that for Japanese national data (Fig. 2). This result may reflect that immigrants are not at risk for mood disorders, which is consistent with a meta-analysis that did not show a significant increase in mood disorders associated with immigration [22].

Immigrants are known to be at risk for substance use for reasons that include acculturative stress, social and economic disparity, and co-morbid mental health disorders. Some reviews have indicated that immigrants, even children and young people, have a high risk of substance use, including drug injection [20, 24, 51]. Actually, the proportion of mental and behavioural disorders arising from psychoactive substance use (ICD-10 code: F1) in this study was higher than that for Japanese national data (Fig. 2).

As mentioned above, the proportion of F4 and F1 were higher than that for Japanese national data; therefore, early intervention, especially for stress-related and substance use disorders, seems to be essential to foreign nationals in Japan.

### Treatment outcome

In terms of treatment continuation, a survey conducted by the World Health Organization showed that the discontinuation rate for psychiatric treatments was about 20% [52]. In a Canadian study of first-episode psychosis, disengagement rates did not differ significantly between immigrant and non-immigrant groups (23% vs. 25%) [53]. Although it remains uncertain whether immigrants are more likely to discontinue treatment than the general population, the discontinuation rate in the present study (32.2%) appeared to be fairly high (Table 1).

### Limitations

Some limitations should be noted in this study. This was a retrospective document review research, and the investigated period was 3 years, which might not be necessarily adequate. Although a retrospective document review has many methodological advantages, difficulties to interpret information, variation in the quality of information and problematic verification are inevitable limitations to some extent [54]. Furthermore, the study sample consisted of data obtained at only three hospitals in the Keihin region; consequently, the characteristics of the hospitals are a potential source of bias.

Detailed epidemiological survey data showing whether foreign nationals are more likely to access central hospitals is not available in Japan. However, these three hospitals are responsible for core areal hospital functions and are the largest hospitals in each district of the Keihin region. In addition, TUO and SYT were accredited by the Japan Medical Services Accreditation for International Patients (JMIP) program [55]. This accreditation system, which was implemented by the Ministry of Health, Labour and Welfare, ensures that international patients can receive Japanese medical services safely and securely. Such accreditation seems to contribute to better access for foreign nationals and referrals from other hospitals and outpatient clinics.

Some people with mental health problems are known to visit physical departments or other departments offering Eastern approaches, including Kampo medicine [56]. As patients visiting these departments were not included in the present study, this could be a limitation.

### Community-based integrated care system and implementation

Based on the above results, further research is needed to reveal how host societies can enrich opportunities for immigrants’ mental health and improve access to social networks for support. There is an international movement toward developing a community-based integrated mental health service, in which mental health professionals and policy makers work together [57–59]. Recent review articles have suggested that an integrated care system for young people was effective for the prevention of mental illness and for early intervention [42]. Some countries have begun to implement school-based programs for supporting the mental health and psychosocial wellbeing of young immigrants [60]. We have undertaken a project named MEICIS (Mental health and Early Intervention in the Community-based Integrated care System), which is funded by the Ministry of Health, Labour, and Welfare of Japan. The present results suggest that an optimal community-based integrated mental health care system that includes early intervention and considers foreign nationals is necessary.

## Conclusions

This study demonstrated one aspect of the current status of foreign nationals with mental health problems in Japan. The results suggest that foreign nationals who reside in Japan are less likely to contact appropriate services for mental illness, especially young people at relatively high risk of mental illness do not access services. Furthermore, lack of medical interpreters may impede the mental health conditions of foreign nationals. The development of a community-based integrated mental health care system accessible to foreign nationals seems to be indispensable.

## Data Availability

The data sets used and /or analysed during the present study are available from the corresponding author on reasonable request.

## Abbreviations

TUO: Toho University Omori Medical Centre
KMH: Kawasaki Municipal Hospital
SYT: Saiseikai Yokohamashi Tobu Hospital
ICD-10: The criteria of the International Statistical Classification of Diseases and Related Health Problems, 10th Revision
